# Clinical impact of a gluten-free diet on health-related quality of life in seven fibromyalgia syndrome patients with associated celiac disease

**DOI:** 10.1186/1471-230X-13-157

**Published:** 2013-11-09

**Authors:** Luis Rodrigo, Ignacio Blanco, Julio Bobes, Frederick J de Serres

**Affiliations:** 1 Gastroenterology, Central University Hospital of Asturias (HUCA), Celestino Villamil, s/n, 33006, Oviedo, Principality of Asturias, Spain; 2 Biomedical Research Office of the Principality of Asturias, FICYT, c/Rosal 7–bis, 33009 Oviedo, Principality of Asturias, Spain; 3 Medicine Department, Psychiatry Area, University of Oviedo, Juan Clavería 6, 33006, Oviedo, Principality of Asturias, Spain; 4 National Institute of Environmental Health Sciences Research Triangle Park, Durham, NC 27709-2233, USA

**Keywords:** Gluten-free diet, Celiac disease, Fibromyalgia syndrome, Irritable bowel syndrome, Health-related quality of life

## Abstract

**Background:**

Celiac disease (CD) is an autoimmune disorder, characterized by the presence of gastrointestinal and multisystem symptoms, which occasionally mimic those of Irritable Bowel Syndrome (IBS) and Fibromyalgia Syndrome (FMS). To assess the effectiveness of a Gluten-Free Diet (GFD) in seven adult female screening-detected CD subjects, categorized as severe IBS and FMS patients.

**Methods:**

All subjects showed villous atrophy in duodenal biopsies, were HLA-DQ2/DQ8-positive, and fulfilled the Rome III and ACR 1990 criteria respectively for IBS and FMS classification. GFD effectiveness was assessed at baseline and after 1 year, examining the score changes in the Tender Points (TPs) test, Fibromyalgia Impact Questionnaire (FIQ), Health Assessment Questionnaire (HAQ), Short Form Health Survey (SF-36), Visual Analogue Scales (VAS) for gastrointestinal complaints, pain and tiredness, drug prescriptions and tissue-Trans-Glutaminase (tTG) serum levels.

**Results:**

At baseline, all patients had poor Quality of Life and VAS scores, a high number of TPs and drug prescriptions, and increased tTG levels. After 1 year of GFD, all outcome measures significantly improved, with a decrease of 51-60% in TPs, FIQ, HAQ, and VAS scales, and in the number of prescribed drugs, accompanied by an increase of 48-60% in SF-36 Physical and Mental Component Summary scores, and a decrease of tTG to normal values.

**Conclusion:**

Results of this pilot study show that the adherence to a GFD by CD-related IBS/FMS patients can simultaneously improve CD and IBS/FMS symptoms, and indicate the merit of further research on a larger cohort.

## Background

Irritable Bowel Syndrome (IBS) is a common gastrointestinal functional disease, characterized by chronic abdominal pain or discomfort, along with diarrhea, constipation (or a pattern of alternation between the two), defecation urgency, tenesmus, bloating and abdominal distension [1,2]. Diagnosis of IBS is based on a positive history of gastrointestinal symptoms (Rome III criteria) in the absence of alarm features, such as: age of onset >50 years, weight loss, rectal bleeding, iron-deficient anemia, systemic signs of infection, or family history of inflammatory bowel disease or colon cancer [3].

Several comorbidities that occur more often in IBS patients than in the general population have been identified, including Fibromyalgia Syndrome (FMS), Chronic Fatigue Syndrome (CFS), Gastroesophageal Reflux Disease (GERD), Temporo-Mandibular Joint (TMJ) disorder, headache, backache, genito-urinary symptoms, anxiety and depression [4,5]. Specifically, FMS, a prevalent chronic widespread pain disorder affecting mainly females (classified according to the 1990 American College of Rheumatology [ACR] criteria) [6], occurs in 20-32% of people with IBS [7]. In turn, 32-70% of people with FMS also meet the criteria for IBS [8,9].

Until about two or three decades ago, Celiac Disease (CD) was considered to be a rare disease, but has now proved to be very common, with a fairly uniform worldwide distribution and an average prevalence of 1-2%. However, it is underdiagnosed, leading to its prevalence being underestimated. Furthermore, the disease can be associated with and/or mimicked by many other intestinal and extra-intestinal diseases [10].

On the other hand, the incidence of CD in IBS patients is more than 7-fold that of the general population [11-13]; for this reason it has been estimated that testing IBS for CD could be cost-effective and therefore recommendable in clinical practice [14].

In recent years, a possible causal link between IBS, FMS and multi-symptomatic forms of gluten sensitivity (CD included) has been suspected on the basis that some cases with overlapping symptoms share similar clinical features [15,16] and that there are some case reports describing isolated patients suffering from FMS and CD simultaneously whose symptoms resolved after removing gluten from their diets [17,18].

The present report refers to a recent active case-finding study for CD conducted by our group among 229 IBS subjects. 104 (45.4%) of these had associated FMS, among whom we detected 7 CD (6.7%) patients. These IBS/FMS/CD-associated patients were started on a gluten-free diet (GFD) and showed a remarkable improvement in their digestive and systemic symptoms on follow-up. Therefore, the main objective of the present report was to analyze the observed clinical, analytical and Health-Related Quality of Life (HR-QoL) changes after 1 year on a GFD in these CD subjects.

## Methods

### Study population and data source

During the 6-year period 2007–2012, we prospectively studied a total of 442 consecutive patients, applying the Rome III criteria for IBS [3] and the ACR 1990 criteria for FMS [6] upon their first visit to the Gastroenterology Outpatient Clinic at the Central University Hospital of Asturias, HUCA (Oviedo, Spain). Most patients were referred from the Rheumatology and Internal Medicine Departments of the same Hospital for the study of a wide variety of long-standing gastrointestinal symptoms.

Diagnosis of IBS was based on a positive history of abdominal discomfort or pain associated with disturbed defecation (Rome III criteria), in the absence of obvious alarm features. Although IBS is no longer a diagnosis of exclusion, since there is good evidence that a positive clinical diagnosis is sufficiently reliable [19], a battery of tests was performed in all patients before commencing the study, a comprehensive medical history was taken, and a thorough physical examination and a complete broad laboratory hematological and biochemical screening were done.

In non-responders to usual therapies, in doubtful cases, or those suspected of having other associated organic illnesses, a specific breath test was performed to exclude possible lactose intolerance or a small bowel bacterial overgrowth. Fecal cultures were performed as needed to rule out parasitic infections in some patients. Furthermore, a total colonoscopy was carried out and random colonic biopsies were taken of patients with persistent diarrhea to rule out microscopic colitis.

An immunological fecal occult blood test (iFOBT) was done in patients aged over 50 years and in those with a positive familial history of colon cancer in first-degree relatives. If this iFOBT was positive, the study was completed with a total colonoscopy.

The inclusion criteria for IBS patients were: (1) Age 18 years or over but less than 65 years; (2) Positive Rome III criteria; (3) Absence of any other associated organic gastrointestinal disease; (4) Negative ACR 1990 criteria for FMS. The same criteria were used to select IBS patients with associated FMS, except the last one, since, by definition, patients had to fulfill the ACR 1990 for FMS classification criteria.

The exclusion criteria for IBS were: (1) Age under 18 or above 65 years; (2) Incomplete or doubtful Rome III criteria; (3) Incomplete or doubtful ACR 1990 criteria for IBS/FMS subjects; (4) Presence of any organic gastrointestinal disease; (5) Any abnormal finding in the analytical screening, colonoscopy or colonic biopsies; (6) Presence of alarm signs or symptoms; (7) Unwillingness to participate in the study.

A total of 263 out of 442 individuals (59%) were eligible to participate in the study. Thirty-four declined to sign the written consent and were excluded. In the end, 229 subjects agreed to take part in this study. Participants were then assigned to one of two groups: the IBS plus FMS group, comprising 104 patients who fulfilled both the Rome III criteria for an IBS diagnosis and the ACR 1990 criteria for FMS classification; the IBS group, comprising the 125 age- and sex-matched, unrelated patients from the same Asturian population who fulfilled the Rome III criteria for IBS diagnosis, but did not exhibit FMS widespread pain or tender points on the skin, and who had negative ACR 1990 criteria.

These 229 subjects were invited to participate on a voluntary basis, after signing a specific informed consent form. The Study Project was approved by the Research and Ethics Committee of the HUCA, following the principles included in the modified Declaration of Helsinki.

### Outline of the study protocol

Each volunteer underwent a clinical evaluation, including an updated medical history, a thorough physical examination, a Health-Related Quality of Life (HR-QoL) battery test, and a broad analytical panel.

### Duodenal biopsy studies

An upper gastrointestinal endoscopy with at least 4 duodenal biopsies was performed in all the patients included in this case finding/screening, following the usual methodology employed in our Service for CD diagnosis. Samples were routinely stained with Hematoxylin-Eosin (HE) and anti-CD3 immunohistochemical monoclonal antibodies to verify the presence and to account for the number of intraepithelial lymphocytes (IELs). These were in turn quantified per 100 epithelial cells. Sam-ples were studied by two expert pathologists from the HUCA and classified into the following types: Stage 0: Histologically normal duodenum; Stage 1: Increased IEL infiltration with a total count ≥ 25% epithelial cells; Stage 2: Crypt hyperplasia and diffuse chronic inflammatory infiltrate of the lamina propria; Stage 3: Villous atrophy, subdivided into three categories: a) mild, b) moderate, and c) severe, according to the histological classification for CD described by Marsh in 1992 [20] and modified by Oberhüber et al. [21].

### Tender points (TPs)

TPs were identified and quantified by digital pressure on the 18 anatomical locations recommended by the ACR 1990 study [6].

### Physical, mental, psychological, social functioning and quality of life questionnaires

To measure their physical, mental, psychological, and social functioning, each patient filled out the self-administered Spanish version forms of the Fibromyalgia Impact Questionnaire (FIQ), the Health Assessment Questionnaire (HAQ), and the Short Form Health Survey (SF-36).

#### FIQ metric

This 10-item instrument measures physical functioning, work status, depression, anxiety, sleep, pain, stiffness, fatigue, and wellbeing on a rating-structured questionnaire, yielding scores between 0 and 80 points. Total FIQ scores are used to define FMS severity, with scores of 0–39, 40–59, and 60–80 corresponding to mild, moderate, and severe FMS, respectively [22].

#### HAQ metric

The 20-item disability scale of the HAQ measures a patient’s difficulty and need for help and assistive devices in activities of daily living. Each item is scored on a 4-point scale, the highest representing maximum impairment: 0 = able to do without any difficulty, 1 = some difficulty, 2 = much difficulty, and 3 = unable to do [23].

#### SF-36 metric

The 36 multi-item scale of the short form SF-36 covers 8 aspects of physical and mental health: (1) Physical functioning (PF); (2) Role physical (RF); (3). Bodily pain (BP); (4) General health (GH); (5) Vitality (VT); (6) Social functioning (SF); (7) Role emotional (RE); (8) Mental health (MH).

The scores of these 8 items are aggregated into two weighted, norm-based Physical and Mental Component Summaries (PCS and MCS, respectively), which take values from 0 (poorest health) to 100 (best health status). No reliable relationship has been established between presence and severity of disease and SF-36 scores. For guidance, the published mean values (and standard deviations) for adults in the general Spanish population are 73.0 ± 27.8 (PCS) and 74.4 ± 24.4 (MCS) [24].

To evaluate the severity of digestive symptoms and the amount of pain and fatigue experienced by the patients we used three appropriate types of Visual Analogue Scales (VAS) [25-27].

Cell blood count and coagulation studies were performed with an automated Abbott Hematology Analyzer (Cell Dyn 3500), and the Coagulation Analyzer ACL 3000 (Menarini), respectively. Biochemical tests were performed on a Hitachi Modular automated analyzer SXA-PPBD (Roche) using enzymatic or kinetic methods to measure urea, glucose, total protein, albumin, C-reactive protein, calcium, folate, vitamin B-12, creatinine, creatine kinase, rheumatoid factor, lipid profile, liver function, IgG, IgA and IgM immunoglobulins, iron metabolism, thyroid function, and to carry out a urinalysis with microscopic examination of the sediment.

Anti-nuclear antibodies (ANAs) and anti-thyroid peroxidase (anti-TPO) antibodies were measured in each participant. In cases with altered liver function test results, anti-mitochondrial antibodies (AMAs) were assessed by indirect immunofluorescence assay in the Hep-20-10 cell line (Euroimmun, Lübeck, Germany).

Anti-IgA tissue Trans-Glutaminase subtype 2 (tTG) antibodies were measured with an ELISA kit from Phadia Diagnostics (Upsala, Sweden).

HLA-DQ2 genetic markers (DQA1*0501 and DQB1*0201 alleles) were determined by a polymerase chain reaction (PCR) with a Protrans® HLA Celiac Disease Domino System (Protrans, Ketsch, Germany) kit. The HLA-DQ8 haplotype was characterized in an HLA-DQ2-negative case with villous atrophy.

##### Statistical analysis

Descriptive statistics were derived for continuous variables (mean, standard deviation, and range) and categorical variables (percentages). Normally distributed continuous outcome measures were analyzed using Student’s t tests, or ANOVA followed by a *post hoc* Fisher’s test, as appropriate. The chi square contingency test (or Fisher’s exact test where appropriate) was used to analyze categorical data. All statistical tests were carried out using SPSS 15.0 (SPSS Inc, Chicago, IL, 2009). Two-sided P values < 0.05 were considered to be statistically significant.

## Results

Of the 229 patients, 125 (54%) were diagnosed with IBS; 104 were female (84%), and the mean age was 51 ± 8 years. The mean duration of symptoms was 29 ± 5 years. Based on stool consistency, they were divided into 3 groups: 74 with constipation, 33 with diarrhea, and 18 mixed.

The remaining 104 (46%) were diagnosed with IBS + FMS; 93 were female (89%), with a mean age of 50 ± 8 years. The mean duration of symptoms was 29 ± 7 years. Distribution with respect to stool consistency was as follows: 68 with constipation, 25 with diarrhea, and 9 mixed. No clinical differences were observed between the two groups.

Seven of the 104 IBS + FMS patients were diagnosed with CD (7%). All were females, with a mean age of 49 ± 12 years (range, 34–68 years). Increased serum levels of tTG, were present in all patients, with mean values of 60 ± 52 U/ml (range, 12–150 U/ml). Genetic markers of CD susceptibility were positive in all cases (HLA-DQ2 in 6, and HLA-DQ8 in 1 case).

From a total of 104 FMS patients, the histopathological analysis of duodenal biopsies revealed 58 cases (56%) showing features of lymphocytic enteritis corresponding to a Marsh type 1 lesion.

Mild-to-moderate villous atrophy (Marsh 3a-3b) was observed in the duodenal biopsies in all CD cases (7%). A positive familial history of CD was found in two cases, and of FMS in one case.

Finally, we found 39 cases (37%) presenting either minimal histological changes or a completely normal duodenal histology.

The seven CD patients exhibited a combination of the following gastrointestinal symptoms: diffuse abdominal pain/discomfort, constipation, diarrhea, alternating diarrhea/constipation, bloating and heartburn. In the vast majority of cases, patients started noticing these symptoms in their 20s. In addition, all complained about a number of common FMS symptoms, including widespread soft-tissue pain, abnormal fatigue, sleep disturbances, cognitive dysfunction, etc. The mean duration of FMS-related symptoms was 7 ± 4 (range, 4–15) years.

All patients had very poor HR-QoL profiles, as measured by FIQ, HAQ and SF-36 tests, and were consequently categorized as severely affected FMS patients.

In addition, they presented a number of other associated diseases, including osteoporosis and temporo-mandibular joint disorders (TMJs).

All patients had been taking several drugs, predominantly analgesics, proton-pump inhibitors (PPIs), anti-depressants and anxiolytics for a long time.

Hematological and general biochemical analyses were within normal ranges in all seven patients at the time of inclusion.

After 1 year of GFD, all the selected outcome measure scores (TPs, FIQ, HAQ, SF-36; VAS for gastrointestinal, complaints of pain and tiredness, and prescribed drugs for symptom control) improved over 50% with respect to baseline (P < 0.001), and the serum concentrations of tTG decreased substantially until normalization in all cases (P < 0.05). TPO and AMAs steadily dropped to normal values in all cases as well (Table 1).

**Table 1:** Comparison of global outcome measures, comparing basal (pre) and after one year (post) on a GFD: Change is calculated as the percentage improvement in scores

	***Pre-GFD***	***Post-GFD***	**Change (%)**	***P***
TPs (scale 0–18)	16.3 (2.4)	8.0 (1.6)	8.3 (51)	< 0.001
FIQ (scale 0–80)	74.3 (2.9)	36.6 (4.0)	37.7 (51)	< 0.001
HAQ (scale 0–3)	1.7 (0.6)	0.7 (0.3)	1.0 (52)	< 0.001
VAS digestive (scale 0–100)	47.8 (2.4)	19.1 (0.8)	28.7 (60)	< 0.001
VAS pain (scale 0–10)	8.0 (0.5)	3.9 (1.0)	4.1 (52)	< 0.001
VAS fatigue (scale 0–10)	7.9 (0.3)	3.9 (0.8)	4.0 (51)	< 0.001
SF36-PCS (scale 0–100)	27.4 (3.6)	40.4 (5.1)	+13.0 (48)	< 0.001
SF36-MCS (scale 0–100)	17.4 (2.9)	27.8 (3.3)	+10.4 (60)	< 0.001
Prescribed drugs (N)	6.6 (1.5)	3.3 (0.5)	3.6 (55)	< 0.001
tTG2 (U/ml)	60.4 (52.2)	0.7 (0.2)	59.7 (98)	< 0.05

Data are expressed as mean and standard deviation. *TPs* Tender Points. *FIQ* Fibromyalgia Impact Questionnaire. *HAQ* Health Assessment Questionnaire. *VAS* Visual Analogue Scale. *SF-36* Short Form Health Survey; *PCS* Physical Component Summary; *MCS* Mental Component Summary. *N* number. *tTG-2* Tissue Trans-Glutaminase-2. P < 0.05, significant; P < 0.001, highly significant.

**Figure 1:**
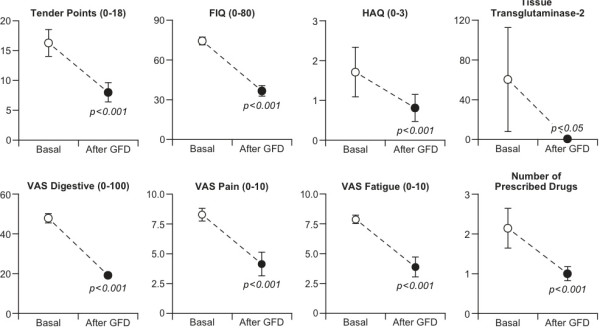
Changes in outcome measures, comparing baseline and after one year of GFD.

Mean changes and standard deviations observed in the different measures of HR-QoL included in the study, before and after a GFD, are shown in (Figure 1).

**Figure 2:**
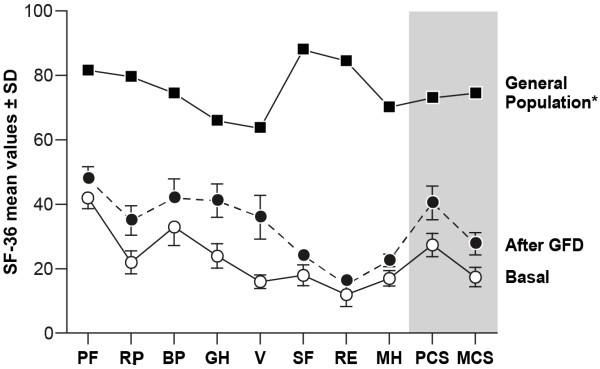
**Comparison of mean SF-36 scores for patients at baseline and after one year of GFD compared with those in the healthy general Spanish population **[24]**. ***Abbreviations:* PF, Physical Functioning; RP, Role Physical; BP, Bodily Pain; GH, General Health; VT, Vitality; SF, Social Functioning; RE, Role Emotional; MH, Mental Health; PCS, Physical Component Summary; MCS, Mental Component Summary.

We also found a clear improvement in the individual and global components of the SF-36 scores when comparing the baseline values with the results obtained 1 year after the introduction of the GFD, although the values did not reach those observed in the general Spanish population (Figure 2).

## Discussion

The most relevant finding of the present study was the remarkable improvement achieved for all outcome measures after one year of uninterrupted GFD in 7 CD females previously categorized as severe IBS/FMS patients recruited through case-finding among IBS and FMS patients.

Curiously, the clinical presentation of these “IBS/FMS CD-associated” patients exhibited a repetitive chronological sequence, usually starting as gastrointestinal relapsing disorders, frequently misclassified as IBS, when the patients were in their 20s and 30s, becoming multisystem “FMS-like” complaints around their mid-40s. Notably, about 30% of these patients mentioned having other first-degree relatives diagnosed with CD. In addition, they scored very poorly in TPs and HR-QoL tests, and as this negative clinical picture would suggest, these patients reported not only frequent use of healthcare services and multiple drug prescriptions, but also low rates of medication adherence due to drug intolerance, lack of effectiveness, or drug-related adverse effects. Finally, an excess of associated diseases and a high prevalence of circulating autoantibodies were documented.

After 1-year of sustained GFD, a very significant decrease (ranging from 51 to 60%) in TPs, FIQ, HAQ and VAS mean scores, and a positive change of 48% and 60% in the SF-36 physical and mental scores, respectively, were observed. For example, the FIQ mean score decreased from more than 60 to less than 39 (i.e., from “severe” to “mild” FMS); the mean HAQ score category changed from “much difficulty” to “able to do with some difficulty”; the VAS for digestive symptoms changed from “moderate/severe” to “slight/moderate” intensity; the VAS for the amount of pain changed from “very bad” to “not too bad”, and a similar result was obtained for the amount of fatigue patients felt. Alongside this clinical improvement, a significant decrease of 55% in both the number and doses of prescribed drugs was reported, and interestingly, the unpleasant manifestations of some associated diseases, such as chronic urticaria, improved significantly. On the other hand, the basal tTG-2, TPO and AMA serum levels steadily dropped to normal values in all patients.

CD is a multisystem autoimmune disorder related to a permanent intolerance of gluten (a protein found in bread, pasta, cookies, pizza crust and many other foods containing wheat, barley or rye) that affects 1-2% of the population (mainly females) worldwide, with subjects generally being carriers of one of the two HLA-II genotypes, DQ2 and DQ8. In these subjects, gliadin peptides trigger an aberrant immune response, resulting in the production of tTG autoantibodies, and an immune-mediated chronic inflammation of the small bowel mucosa, characterized by villous atrophy, intraepithelial lymphocytosis, and crypt hyperplasia. Clinical manifestations of CD may appear at any age, with gastrointestinal and/or extra-intestinal systemic symptoms, although some diagnoses can be made in asymptomatic individuals. Notably, a gluten-free diet (GFD) results in complete clinical remission and full recovery of the intestine in the vast majority of CD patients [28,29].

On the other hand, FMS is a complex chronic pain syndrome affecting around 2% of the population (mainly females) worldwide, characterized by widespread soft-tissue pain, generalized tender points, abnormal fatigue, non-restorative sleep, and a variety of additional symptoms. Its pathogenesis remains elusive, and no analytical test or imaging techniques for objective diagnosis are currently available. Thus, an exclusion diagnosis must be used when other typified diseases are ruled out after an appropriate evaluation study in patients fulfilling the American College of Rheumatology (ACR) 1990 criteria for FMS [6]. Another troubling aspect of FMS is the lack of effective therapy for controlling its symptoms, which makes it a major source of personal, family and social disturbances, and leads to heavy use of health care services, increased work absenteeism, disability and early retirement [30-32].

The striking results of the present trial suggest that a triggering gluten-related autoimmune inflammatory process within the gastrointestinal tract may end up contributing to the onset or increasing the well-documented central nervous system sensitivity responsible for FMS disorder in some CD- or gluten-sensitive individuals [30]. This hypothesis appears to be consistent with the increased prevalence of FMS described in women with different chronic inflammatory processes within the gastrointestinal tract [31,32], and with the fact that our patients reported a long-term history of gastrointestinal complaints preceding the onset of generalized FMS symptoms by decades. Specifically, the comorbid triad of IBS, chronic fatigue and musculoskeletal pain has been considered striking, and other authors have suggested that it may point to an underlying common food hypersensitivity-related mechanism [15].

ANAs were positive in three cases, but none fulfilled the ACR 1982 criteria required for a diagnosis of Systemic Lupus Erythematosus [32]. High serum levels of anti-TPO were found in two cases with minor and transitory thyroid function test result alterations, and AMA antibodies were positive in one case presenting mild elevations of serum liver enzymes without clinical manifestations of liver disease.

Autoantibodies are a characteristic feature of CD, and it is thought that they contribute to the extra-intestinal organ involvement by attacking multiple body cells, finally causing inflammatory damage through an antigen-antibody interaction at the vascular level. In fact, 75% of CD patients have anti-TG2/TG3/TG6 circulating antibodies in blood vessels [33,34], and although anti-tTG2 antibodies are primarily deposited in the intestine, they have also been found in a diversity of other tissues such as skin, oral mucosa, muscle, liver, cerebellum, lymph nodes, etc., whereas anti-gliadin antibodies have been reported to bind to Purkinje cells and dorsal root ganglion neurons [35,36].

The findings of the present trial may have some biases arising from the small number of patients involved and from the lack of reliable biomarkers for IBS and FMS diagnosis/follow-up (compulsorily based on questionnaires, which are inevitably subjective). Our results are nevertheless promising, because apart from providing new research perspectives on IBS and FMS, a relatively poorly understood area of scientific knowledge, the detection of misdiagnosed CD-related IBS/FMS cases would lead to the implementation of a GFD, a simple therapeutic action that could kill two birds with one stone by simultaneously improving gastrointestinal and extra-intestinal symptoms. Furthermore, in the long term, it could prevent further CD-associated complications in patients and undiagnosed relatives at risk of developing overt CD.

## Conclusions

In IBS/FMS patients, the varied extra-intestinal clinical presentations sometimes make CD diagnosis challenging. It is therefore important for clinicians of all subspecialties to be aware of the diverse symptoms and diseases associated with CD so they can consider, at very least, performing serological tests for celiac disease. When these are positive, it would also be useful to perform appropriate HLA tests and duodenal biopsies before proceeding with a gluten withdrawal diet in these patients.

## Abbreviations

CD: Celiac disease; FMS: Fibromyalgia syndrome; GFD: Gluten-free diet; TPs: Tender points; FIQ: Fibromyalgia impact questionnaire; HAQ: Health assessment questionnaire; VAS: Visual analogue scale; tTG: Tissue trans-glutaminase; HR-QoL: Health-related quality of life; HLA: Human leukocyte antigens; ACR: American college of rheumatology.

## Competing interests

The authors declare that they have no competing interests.

## Authors’ contributions

LR, study design, diagnosis and review of patients, analysis of results, and initial and final revision of the manuscript; IB, study design, analysis of results and initial and final writing of the manuscript: JB, study design, analysis of HR-QoL results, and initial and final review of the manuscript; FdeS, valuable suggestions about the study design, analysis and interpretation of results, review of the scientific content and approval of the English language of the final manuscript. All authors read and approved the final manuscript.

## Pre-publication history

The pre-publication history for this paper can be accessed here:

http://www.biomedcentral.com/1471-230X/13/157/prepub
